# In vivo base editing of post-mitotic sensory cells

**DOI:** 10.1038/s41467-018-04580-3

**Published:** 2018-06-05

**Authors:** Wei-Hsi Yeh, Hao Chiang, Holly A. Rees, Albert S. B. Edge, David R. Liu

**Affiliations:** 1 Department of Chemistry and Chemical Biology, Harvard University, Cambridge, MA 02138 USA; 2 Howard Hughes Medical Institute, Harvard University, Cambridge, MA 02138 USA; 3 Merkin Institute of Transformative Technologies in Healthcare, Broad Institute of MIT and Harvard, Cambridge, MA 02142 USA; 4 Eaton-Peabody Laboratory, Massachusetts Eye and Ear, Boston, MA 02114 USA; 5 Program in Speech and Hearing Bioscience and Technology, Harvard Medical School, Boston, MA 02115 USA; 6 Department of Otolaryngology, Harvard Medical School, Boston, MA 02115 USA; 7 Harvard Stem Cell Institute, Cambridge, MA 02138 USA

## Abstract

Programmable nucleases can introduce precise changes to genomic DNA through homology-directed repair (HDR). Unfortunately, HDR is largely restricted to mitotic cells, and is typically accompanied by an excess of stochastic insertions and deletions (indels). Here we present an in vivo base editing strategy that addresses these limitations. We use nuclease-free base editing to install a S33F mutation in β-catenin that blocks β-catenin phosphorylation, impedes β-catenin degradation, and upregulates Wnt signaling. In vitro, base editing installs the S33F mutation with a 200-fold higher editing:indel ratio than HDR. In post-mitotic cells in mouse inner ear, injection of base editor protein:RNA:lipid installs this mutation, resulting in Wnt activation that induces mitosis of cochlear supporting cells and cellular reprogramming. In contrast, injection of HDR agents does not induce Wnt upregulation. These results establish a strategy for modifying posttranslational states in signaling pathways, and an approach to precision editing in post-mitotic tissues.

## Abstract (web summary)

Base editing allows the precise introduction of point mutations into cellular DNA without requiring double-stranded DNA breaks or homology-directed repair, which is inefficient in postmitotic cells. Here the authors demonstrate in vivo base editing of post-mitotic somatic cells in the postnatal mouse inner ear with physiological outcomes.

## Introduction

Standard genome editing agents such as ZFNs, TALENs, or Cas9 are programmable nucleases that induce a double-stranded DNA break (DSB) at the target locus^1–4^. While such agents can efficiently disrupt genes by inducing non-homologous end joining (NHEJ) and other processes that result in stochastic insertions and deletions (indels) and translocations at the site of interest, the introduction of precise changes such as point mutations in genomic DNA using homology-directed repair (HDR) is difficult. Recutting of edited DNA containing a single point mutation can substantially erode yields of desired product^5^. In addition, HDR is thought to be restricted primarily to the S and G2 phases of the cell cycle, when homologous recombination between sister chromatids naturally takes place^6^. Since most post-mitotic cells poorly express the cellular machinery required for this process, HDR in post-mitotic cells is typically very inefficient^1,7,8^.

We recently developed base editing, an alternative genome editing strategy that directly converts one base pair to another base pair at a target locus without reliance on HDR and without introducing double-stranded DNA breaks that lead to an abundance of indels^3,9–11^.The most widely used base editors are fusions of a catalytically disabled form of Cas9, a cytidine deaminase such as APOBEC1, and a DNA glycosylase inhibitor such as uracil glycosylase inhibitor (UGI)^3^. Third-generation base editors (BE3 and its variants) convert C•G base pairs to T•A base pairs at programmable target loci within a window of ~1–5 nucleotides and are compatible with a wide variety of protospacer-adjacent motif (PAM) sequences^10^. A new class of adenine base editors using a laboratory-evolved deaminase domain convert A•T to G•C base pairs with minimal byproducts^9^. Base editing has proven to be a robust approach to achieving efficient, permanent conversion of individual base pairs with minimal indel formation in fungi, plants, mammalian cells, zebrafish, mice, frogs, and even human embryos^10,12–19^.

The steps involved in base editing are not thought to rely on cellular recombination machinery^3,9^, raising the possibility that the process might take place efficiently in non-dividing cells in vivo. We sought to test the ability of base editing, compared with a current HDR method, to generate precise point mutations in terminally differentiated cells in vivo efficiently enough to result in a physiological outcome. In the mammalian inner ear, sensory cells such as cochlear supporting cells and hair cells are post-mitotic^20^. The apparent lack of sensory cell regeneration in the mammalian cochlea contributes to progressive, permanent hearing loss after damage. Recent studies in transgenic mice suggest that stabilization of β-catenin protein can facilitate the regeneration of sensory hair cells by increasing signaling through the canonical Wnt pathway^21,22^. Activation of Wnt signaling stimulates the proliferation of supporting cells and can induce the development of hair cells from supporting cells^23^, suggesting that stabilization of β-catenin in the cochlea might trigger similar cellular reprogramming events, even though additional steps are likely needed for these cells to become functional hair cells^24,25^.

**Fig. 1:**
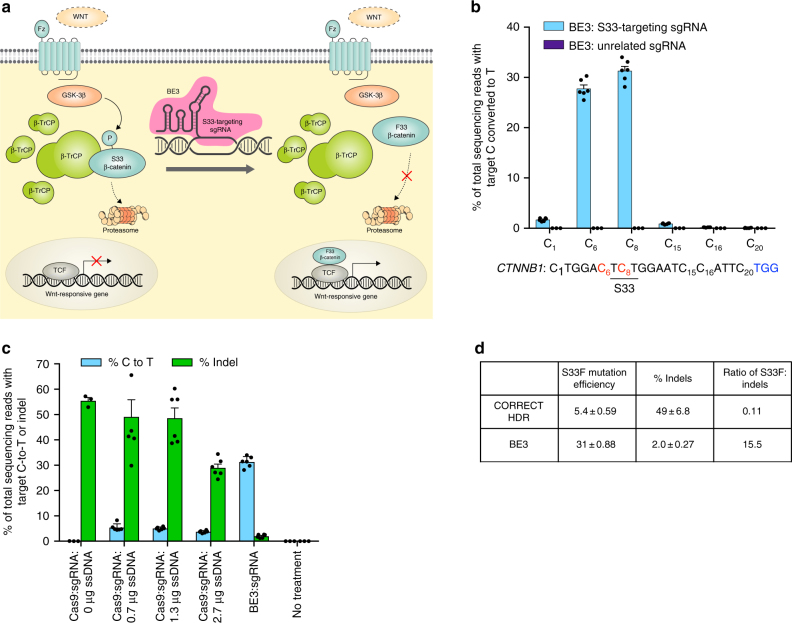
Base editing strategy and comparison of HDR and base editing following plasmid delivery. **a** Schematic representation of the canonical Wnt pathway and a base editing strategy to stabilize β-catenin. In the absence of Wnt signaling, β-catenin is phosphorylated at Ser 33 by GSK-3β and degraded in a phosphorylation-dependent manner. Base editing with BE3 precisely mutates the Ser 33 codon to instead encode Phe, which cannot be phosphorylated. The resulting S33F β-catenin has an extended half-life and can activate target gene transcription by binding with TCF/LEF transcription factors. **b** HEK293T cells were transfected with plasmids expressing BE3 and S33-targeting sgRNA, or BE3 and an unrelated sgRNA. The percentage of total sequencing reads (with no enrichment for transfected cells) with C8 converted to T8 (resulting in the S33F mutation) was measured with high-throughput sequencing (HTS). **c** Plasmid delivery of Cas9 and BE3 (750 ng) with S33-targeting sgRNA (250 ng) into HEK293T cells using 1.5 µL of Lipofectamine 2000 per well of a 48-well plate. C-to-T conversion efficiency and **d** product selectivity ratio (desired S33F mutation: undesired indel ratio) resulting from the best-performing ratio of Cas9:sgRNA:ssDNA template and BE3. Values and error bars reflect mean ± S.E.M. of three biological replicates performed on separate days

Wnt activation induces β-catenin accumulation in the cytoplasm and translocation into the nucleus, resulting in the activation of Wnt target genes. In the absence of Wnt activation (Fig. 1a), cytosolic β-catenin is phosphorylated at specific serine and threonine residues by glycogen synthase kinase 3β (GSK-3β)^26^. Phosphorylated β-catenin is recognized by β-transducin repeat-containing proteins (β-TrCP), resulting in the ubiquitination and degradation of β-catenin (Fig. 1a)^27^. Previously a small-molecule GSK-3β inhibitor and histone deacetylase inhibitor were used to upregulate Wnt-responsive genes, resulting in substantial expansion of supporting cells and differentiation into hair cells in vitro^28^. However, toxicity arising from inhibition of protein kinases that share homology with GSK-3β^29^ as well as the potential for oncogenesis from widespread upregulation of Wnt activity^30,31^ limits the use of small-molecule GSK-3β inhibitors in vivo.

To test the ability of in vivo base editing in post-mitotic cells to induce a physiological outcome, we hypothesize that we could upregulate Wnt activity in the inner ear by stabilizing β-catenin through base editing. β-catenin lacking phosphorylation at Ser 33 or Ser 37 is stabilized since it is not ubiquitinated by β-TrCP^32–34^. Deletion of β-catenin exon 3, which contains the sites of GSK-3β-mediated phosphorylation, causes supporting cell proliferation and hair cell transdifferentiation in the postnatal sensory epithelium of transgenic mice^21,22,35^. However, unless exon 3 deletion is limited to the inner ear, the resulting mice develop tumors in a variety of organs, including liver, prostate, and skin^36^. Local delivery of a base editor to prevent β-catenin phosphorylation in the cochlea, which shows a striking resistance to oncogenesis^37^, may enable supporting cell renewal and hair cell generation.

In this study we develop a base editing strategy to alter protein posttranslational modification and stability in post-mitotic cells in vivo. We use BE3 to recode a single residue in the gene encoding β-catenin, preventing phosphorylation and degradation of its protein product. In cell culture, base editing of β-catenin increases its abundance and elevated Wnt signaling. In the cochlea of postnatal mice, lipid-mediated delivery of the β-catenin-targeting base editor results in the proliferation of post-mitotic supporting cells and the differentiation of supporting cells into cells expressing the hair cell marker Myo7a. Our findings establish that base editing can be used to change the posttranslational modification state, abundance, and signaling potential of a protein in post-mitotic cells in vivo, and also suggest an approach to localized Wnt signaling activation.

## Results

### Base editing prevents phosphorylation of Ser 33 in β-catenin

We hypothesized that base editing the β-catenin gene (*CTNNB1* in humans) to replace an amino acid that is phosphorylated by GSK-3β with a residue that cannot be phosphorylated would impede β-catenin degradation and increase activation of T-cell factor/lymphoid enhancer factor (TCF/LEF) transcription factors. Previous work suggests that mutation of β-catenin Ser 33 to Tyr, Pro, or Cys can abolish recognition by β-TrCP, preventing degradation of β-catenin^33^. We designed a single-guide RNA (sgRNA) that when complexed with BE3, should introduce a C-to-T mutation at the DNA nucleotide encoding Ser 33 in β-catenin, resulting in the conversion of a TCT (Ser) codon to a TTT (Phe) codon (Fig. 1b). Murine S33-targeting sgRNA and human S33-targeting sgRNA share very similar protospacers and both target the same codon. Since the phenylalanine side chain cannot be phosphorylated, this change should increase the cytosolic lifetime of β-catenin, increasing activation of TCF/LEF transcription factors and downstream signaling (Fig. 1a).

Next we tested if base editing is capable of installing the desired point mutation into β-catenin in cultured human cells. We transfected plasmids expressing BE3 and S33-targeting sgRNA or an unrelated control sgRNA into HEK293T cells. After a 3-day incubation, cells were harvested and the C-to-T conversion efficiency at nucleotide C8 of the β-catenin Ser 33 codon (counting the PAM as nucleotide positions 21–23) was measured by high-throughput DNA sequencing (Fig. 1b). We observed efficient mutation of the target codon from TCT to TTT (31% ± 0.9%) together with the efficient conversion of another cytosine within the base editing window, C6–T6 (28% ± 0.7%) (all efficiencies listed are mean ± S.E.M. for three biological replicates with no enrichment for transfected cells). Editing at C6 was expected given the 5-base editing window of BE3 (C4–C8)^3,10^. In addition, C1–T1 (1.7% ± 0.1%) and C15–T15 (0.90% ± 0.05%) conversions were also observed at much lower frequencies, consistent with our previous studies^3,10^. Importantly, unlike editing of the target C8 to T8, conversion of C1, C6, or C15 to T does not change the predicted amino acid sequence of the resulting protein, as all three of the other C-to-T mutations are silent.

We observed low (2.0% ± 0.3%) indel frequencies from BE3 and S33-targeting sgRNA plasmid transfection. Control samples treated with plasmids encoding BE3 and an unrelated sgRNA were also analyzed, resulting in no C-to-T mutation at the target locus above our limit of detection (~0.025% mutation; see Methods). Taken together, these observations validate a base editing strategy that converts the wild-type β-catenin gene to the S33F mutant in mammalian cells efficiently and with a high degree of product selectivity.

### Effect of S33F β-catenin mutation on Wnt signaling in vitro

**Fig. 2:**
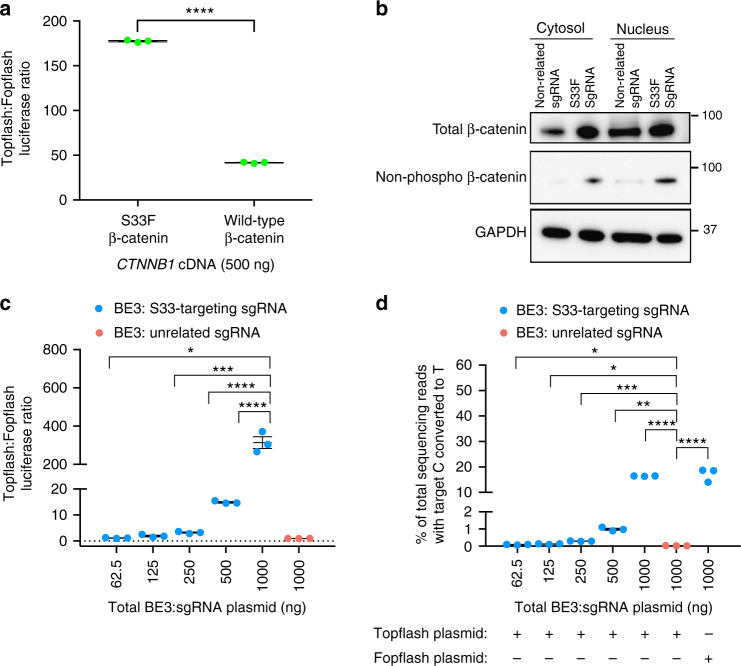
Biological outcomes associated with base editing S33F in β-catenin in human cells. **a** HEK293T cells were transfected with plasmids encoding Topflash (β-catenin-responsive firefly luciferase reporter) or Fopflash (mutant form of Topflash that cannot be activated by β-catenin) and mutant S33F β-catenin or wild-type (Ser 33) β-catenin. Wnt signaling was measured by the ratio of Topflash:Fopflash luciferase activity. **b** Cytosolic and nuclear extracts of HEK293T cells treated with base editor and S33-targeting sgRNA or unrelated sgRNA were subjected to western blot analysis for total β-catenin or non-phosphorylated β-catenin (Ser33/Ser37/Thr41). Each blot represents one antibody. GAPDH was used as a loading control. **c** HEK293T cells were transfected with plasmids encoding the Topflash or Fopflash reporters, base editor, and S33F or unrelated control sgRNA. The Topflash:Fopflash luciferase ratio for BE3 + S33-targeting sgRNA (blue) and BE3 + unrelated sgRNA (red) are shown. **d** Percent C-to-T conversion at the target Ser 33 codon, which results in the S33F mutation in β-catenin, assayed by HTS. Values and error bars reflect mean ± S.E.M. of three biological replicates performed on different days. * *p* ≤ 0.05, ** *p* ≤ 0.01, *** *p* ≤ 0.001, and **** *p* ≤ 0.0001 (Student**’**s two tailed *t*-test)

To test if the S33F mutation in β-catenin increases the amount of non-phosphorylated β-catenin in cells, we measured the amount of total and non-phosphorylated β-catenin using cell fractionation followed by Western blotting (Fig. 2b). We observed 6-fold greater levels of non-phosphorylated β-catenin in nuclear extracts of cells transfected with plasmids encoding BE3 and S33-targeting sgRNA compared to control cells transfected with plasmids encoding BE3 and an unrelated sgRNA (Fig. 2b and Supplementary Fig. 3a). The total amount of β-catenin, including both phosphorylated and non-phosphorylated forms, was 7-fold higher in nuclear protein extracts from cells treated with BE3 and the S33-targeting sgRNA than in control cells treated with BE3 and an unrelated-sgRNA (Fig. 2b and Supplementary Fig. 3a), consistent with stabilization and enhanced translocation of β-catenin into the nucleus.

To assay the effects of installing the β-catenin S33F mutation on Wnt signaling, we used an established Wnt reporter system in HEK293T cells^38^. This assay requires co-transfection with three plasmids: (i) Topflash, which contains TCF/LEF operators that activate expression of firefly luciferase when bound by β-catenin, or Fopflash, a negative control plasmid that contains mutated TCF/LEF sites that cannot be activated by β-catenin; (ii) Renilla, which expresses renilla luciferase to enable normalization for transfection efficiency; and (iii) a β-catenin cDNA plasmid that expresses either the wild-type (Ser 33) or mutated (Phe 33) β-catenin. 3 days post-transfection, the level of TCF/LEF-mediated Wnt signaling was quantified by the luminescence ratio of the Topflash and Fopflash reporters (Fig. 2a). HEK293T cells transfected with a plasmid expressing mutated S33F β-catenin showed a time-dependent increase in the Topflash:Fopflash luminescence ratio (Supplementary Fig. 1c), reaching a maximum ratio of 180 ± 1.2 3 days post-transfection, 4.3-fold higher than the Topflash:Fopflash luminescence ratio in cells transfected with a plasmid expressing wild-type β-catenin (42 ± 0.7) (Fig. 2a). These results support a model in which mutating Ser 33 to Phe in β-catenin increases Wnt signaling activity in mammalian cells.

**Fig. 3:**
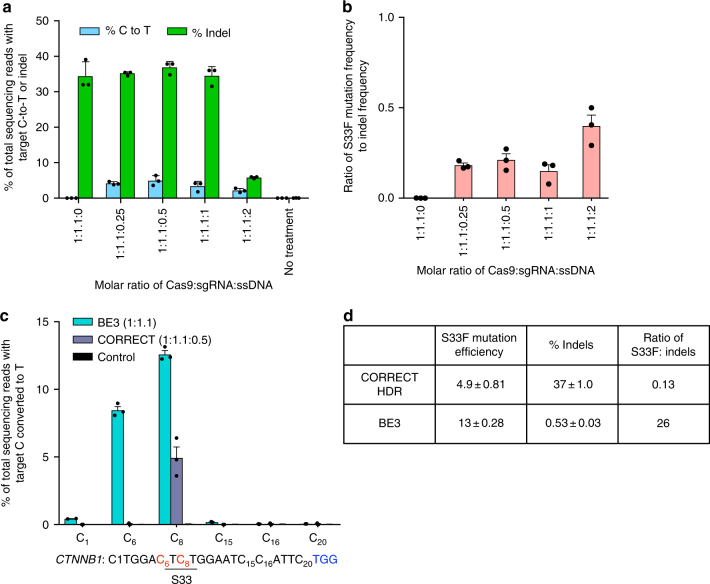
Comparison of efficiency and product selectivity of HDR vs. base editing following RNP delivery. RNP delivery into HEK293T cells of 200 nM Cas9 or 200 nM BE3 pre-complexed with the S33-targeting sgRNA or an unrelated sgRNA and delivered in a cationic liposome. **a** Frequency of S33F mutation (blue) and indels (green) from treatment with CORRECT HDR agents (Cas9, sgRNA, and ssDNA donor template) in the ratios shown. **b** Product selectivity ratio, defined as the ratio of S33F modification to indel modification, resulting from treatment with the CORRECT HDR agents used in **a**. **c** Comparison of target C to T conversion efficiency following RNP delivery of BE3 (blue) or CORRECT HDR (purple) and the S33-targeting sgRNA. The control corresponds to cells treated with BE3 protein and unrelated sgRNA. **d** Product selectivity of base editing and CORRECT HDR. Values and error bars reflect mean ± S.E.M. of three independent biological replicates performed on different days

Next, we used base editing to install the β-catenin S33F mutation in human cells and assayed the effect on Wnt signaling. We co-transfected HEK293T cells with four plasmids: a BE3-expression plasmid, an sgRNA-expression plasmid (either targeting β-catenin Ser 33, or encoding an unrelated control sgRNA), the transfection efficiency reporter Renilla, and either the Topflash or Fopflash reporter plasmid. HEK293T cells transfected with plasmids expressing the reporters and BE3 + S33-targeting sgRNA exhibited a time-dependent increase in Topflash:Fopflash luminescence ratio (Supplementary Fig. 1b). After 3 days, cells treated with the highest dose of base editor (750 ng BE3 plasmid and 250 ng sgRNA plasmid per well of a 48-well plate) exhibited a much higher Topflash:Fopflash ratio (310 ± 31) than cells transfected with BE3 and an unrelated sgRNA (1.0 ± 0.02) (Fig. 2c). The C-to-T conversion efficiency at the target Ser codon was analyzed by high-throughput DNA sequencing (Fig. 2d). At the highest BE3 dose, the C-to-T conversion efficiency at position 33 was 16 ± 0.1% (Fig. 2d). Lower doses of BE3 and sgRNA resulted in markedly lower base editing efficiency and lower Wnt signaling levels (Figs. 2c and 3d). Wnt signaling levels were strongly correlated with S33F base editing efficiency (*R*^2^ = 0.97, *p* < 0.0001 for non-zero slope, linear regression analysis, Supplementary Fig. 1a) suggesting that treatment with BE3 and the S33-targeting sgRNA strongly enhances Wnt signaling levels in a base editing-dependent manner.

Finally, we assayed the ability of β-catenin base editing to increase the expression of known endogenous Wnt-responsive genes in HEK293T cells, including Axin-related protein (*AXIN2*), cyclin D1 (*CCND1*), cyclin dependent kinase inhibitor 1A (*CDKN1A*), and fibroblast growth factor 20 (*FGF20*). Transfection of HEK293T cells with BE3 and β-catenin S33-targeting sgRNA resulted in 1.8 ± 0.21-fold higher expression of *AXIN2*, 1.7 ± 0.17-fold higher expression of *CCND1*, 1.7 ± 0.7-fold higher expression of *CDKN1A*, and 12 ± 2.2-fold higher expression of *FGF20* compared with cells treated with BE3 and an unrelated sgRNA (Supplementary Fig. 3b).

Taken together, these results demonstrate that treatment of cells with the β-catenin S33F base editor converts the endogenous β-catenin gene into the S33F mutant allele, substantially increases levels of non-phosphorylated β-catenin, increases expression of a reporter gene downstream of TCF/LEF operators in a base editing-dependent manner, and also increases expression of endogenous Wnt-responsive genes.

### Comparison of base editing and HDR

A commonly used approach to introduce precise modifications into genomic DNA is to harness HDR using a targeted nuclease and a donor DNA template that contains the desired modification and is homologous to the target locus. We compared the outcomes of base editing to Cas9 nuclease-mediated HDR to install the S33F mutation in β-catenin in HEK293T cells. For this comparison we used the recently described CORRECT HDR method, which introduces desired mutations into target loci more efficiently than previous HDR methods^39, 40^. First we performed CORRECT HDR with a donor DNA template that both installs the desired mutation and that alters the PAM sequence to prevent re-cutting of the desired DNA product. Lipid-mediated plasmid transfection of Cas9:sgRNA constructs and an optimized amount of ssDNA donor template into HEK293T cells resulted in levels of precise installation of β-catenin S33F (5.4 ± 0.6%) and indels (49 ± 7%) consistent with previous reports^40^ (Fig. 1c). Since plasmid transfection of BE3:sgRNA constructs resulted in 31 ± 0.9% conversion of β-catenin Ser 33 to Phe and 2.0 ± 0.3% indels (Fig. 1d), the product selectivity ratio (desired S33F mutation:undesired indel ratio) was 0.11 for CORRECT HDR and 16 for base editing, a 140-fold difference.

We recently demonstrated delivery of Cas9 nuclease ribonucleoprotein (RNP) complexes to the inner ear to mediate spatially localized genome editing in vivo with enhanced DNA specificity relative to plasmid transfection^11,41,42^. In light of these advantages, we also compared RNP delivery of base editors with RNP delivery of HDR agents. We optimized RNP delivery-mediated CORRECT HDR by treating cells with different stoichiometries of Cas9 protein, S33-targeting sgRNA, and a donor ssDNA template. Optimized cationic lipid-mediated delivery of Cas9, guide RNA, and donor DNA template resulted in an average HDR efficiency of 4.9 ± 0.8% S33F mutation using a 1:1.1:0.5 molar ratio of Cas9:S33-targeting sgRNA:donor ssDNA template (Fig. 3a). In contrast, the efficiency of installing the S33F mutation using BE3, the same guide RNA, and the same cationic lipid averaged 13 ± 0.3% (Fig. 3c). Consistent with the DNA transfection results above, the CORRECT HDR method was also accompanied by a much higher indel frequency than that of the base editing approach; we observed 37 ± 1% indels from CORRECT HDR, but only 0.52 ± 0.03% indels from base editing (Fig. 3b,d). Therefore, the product selectivity ratio of S33F editing:indels was 200-fold higher for base editing than for CORRECT HDR. Taken together, these results indicate that base editing either by DNA delivery or RNP delivery enables more efficient installation of the S33F mutation in β-catenin with fewer indels and thus much higher product selectivity than the CORRECT HDR method.

### Off-target analysis of β-catenin S33F base editing

Genome editing agents can induce unintended DNA modifications at off-target genomic loci that are similar in sequence to the target locus^43–45^. Recent studies have shown that off-target base editing mediated by BE3 is generally a subset of off-target loci modified by the corresponding Cas9 nuclease and guide RNA, as expected given that the DNA-binding capability of BE3 is derived from Cas9^3,10,11^. We investigated the potential off-target genome editing by Cas9 nuclease programmed by the S33F β-catenin sgRNA used herein with two methods. First, we used GUIDE-Seq^45^, an unbiased genome-wide method that has been extensively used to identify off-target loci in mammalian cells following Cas9:sgRNA exposure (See Methods). We performed GUIDE-Seq on murine NIH/3T3 cells treated with Cas9:S33-targeting sgRNA. The on-target β-catenin locus was identified with 1108 GUIDE-Seq reads, corresponding to an on-target modification frequency of 23%. Despite robust detection of on-target modification, we observed zero GUIDE-Seq reads corresponding to off-target modification following treatment with Cas9:S33-targeting sgRNA (Supplementary Table 1), suggesting little or no Cas9-mediated off-target modification by this guide RNA in NIH/3T3 cells.

As a complementary approach, we used the Cutting Frequency Determinant (CFD) algorithm to predict off-target loci in the mouse genome associated with S33-targeting sgRNA^42,46,47^. Following nucleofection of plasmids encoding Cas9 and S33-targeting sgRNA into NIH/3T3 cells, we performed deep sequencing to measure indel frequency at the top ten computationally predicted off-target loci (Supplementary Table 1). Consistent with the GUIDE-Seq results, we observed no detectable indel formation (<0.05%) at any of the ten predicted off-target loci (Supplementary Table 1).

These data collectively suggest that the S33-targeting sgRNA used to modify the β-catenin locus mediates few, if any, off-target editing events by Cas9 in murine cells. Since off-target base editing is typically a subset of Cas9 off-target modification for a given sgRNA^3,9–11^, these findings suggest that the changes in β-catenin phosphorylation state and Wnt signaling activity are unlikely to arise from off-target base editing.

### In vivo base editing induces post-mitotic cell reprogramming

Base editing relies on cellular mismatch repair machinery, which is expressed in most cells^48,49^, in contrast to the cellular DSB repair and recombination machinery that mediates HDR, which is poorly expressed in non-mitotic cells^50^. This difference raises the possibility that base editing may be effective in post-mitotic cells in vivo, even though HDR in post-mitotic cells remains a major challenge.

We previously discovered that local in vivo injection of cationic lipid reagents normally used for nucleic acid transfection could potently deliver negatively charged proteins or protein:nucleic acid complexes, including Cas9:sgRNA ribonucleoprotein (RNP) complexes, into the organ of Corti^42^. Because activation of Wnt signaling outside the inner ear can promote oncogenesis^51^, such a local delivery platform that minimizes exposure of other cells to the editing agent is ideally suited for in vivo base editing of β-catenin.

**Fig. 4:**
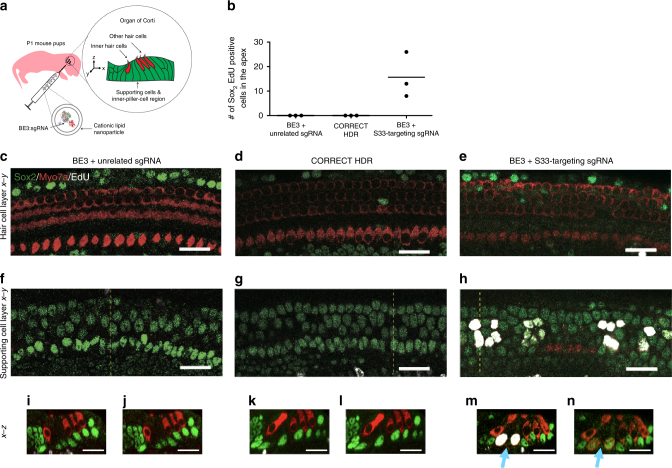
Outcomes associated with in vivo RNP-mediated base editing in post-mitotic cochlea. **a** The cochlea of a postnatal day 1 (P1) mouse was injected with lipid nanoparticles encapsulating BE3 + sgRNA, CORRECT HDR agents, or controls. The next day, mice received 5-ethynyl-2-deoxyuridine (EdU) by subcutaneous injection once per day for 5 days. At postnatal day 7 (P7), one day after the fifth EdU injection, the cochlea was dissected and organ of Corti was visualized by chemical staining (to visualize EdU) and immunofluorescence (Myo7a and Sox2). In the cochlea, Myo7a (red) is expressed in hair cells and Sox2 (green) is expressed in supporting cells. EdU (white) marks newly divided cells. **b** Quantification of Sox2 and EdU double-positive cells at the apical region of the organ of Corti from mice treated with BE3 + S33-targeting sgRNA (*n* = 3), CORRECT HDR agents (*n* = 3), or BE3 + unrelated sgRNA (*n* = 3). Values and error bars reflect mean and S.E.M. **c**–**n** Images from the organ of Corti tissue of mice treated with BE3 + unrelated sgRNA (**c**, **f**, **i**, **j**); CORRECT HDR agents (**d**, **g**, **k**, **l**); or BE3 + S33-targeting sgRNA (**e**, **h**, **m**, **n**). The blue arrows point to triple-positive cells that had undergone proliferation and reprogramming to cells expressing Myo7a. **c**–**e** *x*–*y* plane of hair cell layers. **f**–**h** *x*–*y* plane of supporting cell layers. (**i**, **k**, **m**) *x*–*z* plane of samples in **f**, **g**, **h** at the dotted yellow lines. **j**, **k**, **m** *x*–*z* plane of samples in **f**, **g**, **h** at the dotted yellow lines, but with the Myo7a and Sox2 channels shown. Scale bar (white) = 25 μm

We tested different ratios of base editor:sgRNA:lipid in vivo. The optimal ratio combined purified BE3 (57 µM), the β-catenin S33-targeting sgRNA or an unrelated sgRNA (100 µM) and 2.0 µL cationic lipid in a total volume of 12.0 µL. After a 5-min incubation, we injected 1.0 µL of the resulting mixture into the cochlea of postnatal day 1 (P1) wild-type CD1 mice (Fig. 4a). The intracochlear injection was followed by subcutaneous injection of 5-ethynyl-2′-deoxyuridine (EdU) daily throughout the 5 days to enable detection of proliferating cells. Cochlear tissues were harvested at P7 and EdU, Myo7a (a marker for cochlear hair cells), and Sox2 (a marker for supporting cells) were detected by chemical staining and immunofluorescence.

Confocal microscopy of cochlear tissue harvested from mice treated with BE3 and the β-catenin S33-targeting sgRNA revealed cells positive for both EdU and Sox2, consistent with newly divided supporting cells (Fig. 4h). The post-mitotic status of the postnatal day 7 (P7) cochlear sensory epithelium was confirmed by the lack of EdU incorporation in the organ of Corti, in contrast with EdU incorporation in mesenchymal cells (e.g., tympanic border cells), which are known to be mitotic^22,52,53^ (Supplementary Fig. 2, orange arrowhead). A cochlea treated with BE3 and the β-catenin S33-targeting sgRNA displayed multiple EdU-positive supporting cells in the apical turn (*n* = 3, EdU and Sox2 double-positive cells = 16 ± 5.3, Fig. 4h). We also observed EdU and Sox2 double-positive cells expressing the hair cell marker Myo7a (Fig. 4m,n, blue arrows). Indeed, all EdU and Myo7a double-positive cells observed were also Sox2-positive (Fig. 4e,h,m,n), consistent with transdifferentiation of supporting cells into hair cells^22^. The expansion of supporting cells (EdU and Sox2 double-positive cells) was within the inner pillar cell region and likely represented Lgr5-positive cells, consistent with previous reports^22,28^.

In contrast, treatment of the cochlea with optimized CORRECT HDR reagents (1:1.1:0.5 molar ratio of Cas9: sgRNA: donor ssDNA), resulted in no evidence of newly divided supporting cells (EdU- and Sox2-positive) or newly divided hair cells (EdU- and Myo7a-positive) (Fig. 4d,g,k,l), consistent with the inefficiency of HDR in these post-mitotic cells. A control cochlea treated with BE3 and an unrelated sgRNA also showed no newly divided supporting cells or hair cells (Fig. 4c,f,i,j). The lack of EdU-positive cells in the sensory epithelium excludes the possibility of cell division resulting from lipid-mediated BE3 protein delivery. Together, these results suggest that base editing of Ser 33 to Phe in β-catenin, in contrast with Cas9 nuclease-mediated HDR, can induce cell division and transdifferentiation of supporting cells into hair cells in post-mitotic cells in vivo. This difference can be attributed to mechanistic differences between base editing and HDR-mediated editing, as the cellular machinery that mediates HDR is inactive or poorly expressed in non-dividing cells such as the target cells in the sensory epithelium^50^.

To visualize the location and distribution of cationic lipid-mediated protein and RNP delivery following intracochlear injection into the mouse inner ear of P1 mice, we performed analogous intracochlear injections of lipid complexed with proteins. Cre-mediated recombination in Ai9 tdTomato mice results in tdTomato fluorescence. We injected (–30)GFP–Cre complexed with lipid into these mice, and observed tdTomato fluorescence in supporting cells (Supplementary Fig. 4). We then performed injections into wild-type CD1 mice of lipid complexed with BE3 and fluorescein-labeled S33-targeting sgRNA and observed fluorescein localized within the organ of Corti in regions containing supporting cells and hair cells (Supplementary Fig. 4). Injection of lipid complexed with fluorescein-labeled sgRNA without BE3 did not result in fluorescein signal, likely due to sgRNA degradation. These observations suggest that intracochlear injection of cationic lipid-meditated protein or RNP delivery results in localized delivery within the cochlea, including supporting cells and hair cells of interest.

**Fig. 5:**
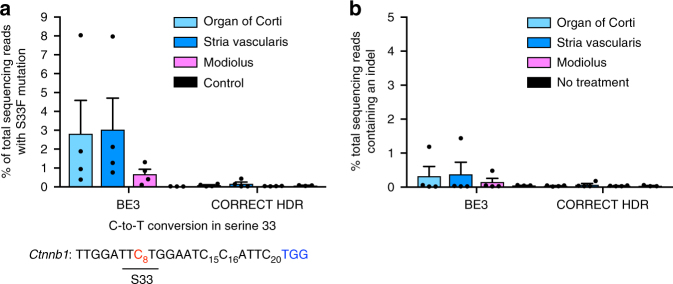
In vivo S33F mutation of β-catenin induced by injection of base editor RNPs. **a** Tissue was harvested from the cochlea of mice injected with either BE3 + S33-targeting sgRNA, or with CORRECT HDR agents. HTS of genomic DNA isolated from tissue samples revealed the frequency of the S33F mutation. Note that because tissue samples contain cells not exposed to editing agents, the observed genome modification frequency in these samples is less than the editing efficiency of treated cells. **b** Indel frequency at the Ser 33 locus following treatments described in **a**. Values and error bars reflect mean ± S.E.M. of four mice injected with BE3 and four mice injected with CORRECT HDR agents. Control samples in **a**, **b** are organ of Corti from three contralateral uninjected ears

High-throughput DNA sequencing of genomic DNA from bulk cochlear tissue of treated mice confirmed base editing of β-catenin Ser 33 to Phe in three regions of the cochlea: the organ of Corti (2.8% of total sequencing reads containing S33F β-catenin), the stria vascularis (3.0%), and the modiolus (0.7%), with low indel formation (averaging 0.4% across all tissues) (Fig. 5). We note that samples of cochlear cells from treated mice included cells that were not exposed to base editor, and thus we expect the percentage of dissected tissue containing the S33F mutation to be substantially less than the frequency of base editing observed in cultured cells, consistent with previous reports^11^. In contrast, we observed no substantial C-to-T conversion (≤0.25%) or indels (≤0.1%) in three regions of the cochlea injected with optimized CORRECT HDR agents (Fig. 5), consistent with the known ineffectiveness of HDR in post-mitotic supporting cells^50^. Collectively, these results confirm that base editing, in contrast to HDR, can mediate local installation of the β-catenin S33F mutation in post-mitotic sensory cells in vivo.

## Discussion

This study establishes in vivo base editing of post-mitotic sensory cells through the local injection of a base editor RNP:lipid complex into the inner ear of mice. Here, the resulting base editing event precisely introduced a S33F mutation into β-catenin, altering its ability to be phosphorylated and decreasing its degradation rate, thereby enhancing Wnt signaling in vitro and in vivo. This single amino acid change was installed in cultured cells more efficiently and with far fewer undesired genome modifications using base editing with BE3 than using HDR with Cas9 nuclease and a donor DNA template. Our observations in vivo also reveal that base editing, but not HDR, can be used to effect physiological changes in the post-mitotic mammalian inner ear, consistent with the lack of dependence of base editing on homologous recombination machinery.

In contrast with the use of base editing to directly restore the sequence of a mutated gene to that of the corresponding wild-type allele^3,9,16,17,54^, this study establishes that base editing the potential of a protein to undergo post-translational modification, in this case to block its ability to be phosphorylated and degraded, can also achieve a desired physiological outcome. Such an approach offers a potential advantage over simple gene correction in cases in which a low level of protein alteration can exert an amplified physiological effect. In this application, the effects of β-catenin S33F base editing, amplified through the ability of a persistent transcription factor to mediate multiple transcriptional events, greatly augments Wnt signaling, leading to detectable changes in cell proliferation and cell state.

Our work establishes that local delivery of base editor as an RNP complex into the cochlea enables a high degree of specificity both for the target DNA locus and for the target cells in the cochlea. These features are distinct from the delivery of diffusible small molecules^20,55,56,57^, which can perturb the activity of homologous protein targets and have a greater potential to affect the homeostasis of other tissues in vivo. Another key feature of the RNP delivery approach used in this study is that precise genome alterations are made without exposing cells to exogenous DNA or virus, preventing the possibility of random integration of DNA into the host cell genome.

Although activating the Wnt pathway in the cochlea is likely insufficient to restore function in a damaged cochlea^57–60^, these findings suggest the potential to manipulate complex signaling pathways by a precise in vivo editing strategy. This approach has potential for in vivo cellular reprogramming, and, in principle, may be applicable to other disorders for which current therapies are repeated dosing of small-molecule Wnt agonists^58,59^.

## Methods

### Animal models

CD1-IGS mice and floxP-tdTomato mice were obtained from Charles River Laboratories. All animal experiments were approved by the Institutional Animal Care and the Use Committee of Massachusetts Eye and Ear.

### Cell line authentication and quality control

HEK293T (American Type Culture Collection, ATCC CRL-3216) and NIH/3T3 (ATCC CRL-1658) were maintained in DMEM plus GlutaMax (Thermo Fisher) supplemented with 10% (v/v) fetal bovine serum, 100 U/mL penicillin/streptomycin (Thermo Fisher) at 37 °C with 5% CO_2_. Cells were obtained from ATCC and were authenticated and verified to be free of mycoplasma by ATCC upon purchase.

### Cloning of plasmids

The sgRNA plasmids were generated by USER cloning. Phusion U Hot Start DNA Polymerase (Thermo Fisher) was used to replace desired protospacers from the sgRNA template plasmid. The cDNA plasmids were generated by site directed mutagenesis (New England Biolabs). Primers were designed with overhang containing the desired point mutation sequence and used to amplify from a previously reported β-catenin cDNA construct^60^. PCR products were carried out using NEB stable Competent cells (New England Biolabs). See Note S2 for a full list of primers used in this study.

### Expression and purification of BE3 protein

BE3 protein was prepared by overexpressing in BL21 Star (DE3)-competent *E. coli* cells using a plasmid encoding the bacterial codon-optimized base editor with a His_6_ N-terminal purification tag. Detailed purification steps are described in our previous study^11^, and the expression plasmid is available on Addgene (Note S1). After protein expression, bacteria cells were lysed by sonication and the lysate was cleared by centrifugation. The cleared lysate was incubated with His-Pur nickel nitriloacetic acid (nickel-NTA) resin. The resin was washed before bound protein was eluted with elution buffer. The resulting protein fraction was further purified on a 5 mL Hi-Trap HP SP (GE Healthcare) cation exchange column using an Akta Pure FPLC. Protein-containing fractions were concentrated using a column with a 100,000 kDa cutoff (Millipore) centrifuged at 3,000 g and the concentrated solution was sterile filtered through an 0.22 μm PVDF membrane (Millipore).

### In vitro transcription of sgRNA

PCR was performed using Q5 Hot Start High-Fidelity DNA Polymerase (New England Biolabs) and primers as listed in the Note S2 to linearize DNA fragments containing the T7 RNA polymerase promoter sequence upstream of the desired 20 bp sgRNA protospacer and the sgRNA backbone. HiScribe T7 Quick High Yield RNA Synthesis Kit (New England Biolabs) was used to transcribe sgRNA at 37 °C for 14–16 h with 1 µg of linear template per 20 µL reaction. Fluorescein-labeled sgRNA was transcribed by adding 10% v/v Fluorescein RNA Labeling Mix (Sigma Aldrich) to the transcription system. Purification of sgRNA was performed with MEGAClear Transcription Clean Up Kit (Thermo Fisher), following the manufacturer’s instructions. Purified sgRNAs (100 µM) were stored in aliquots at −80 °C.

### Protein extraction and western blotting

Total proteins were extracted with radioimmune precipitation assay buffer from whole cells. Cytoplasmic and nuclear protein extracts were prepared with NE-PER nuclear and cytoplasmic extraction reagents (Thermo Fisher) according to the manufacturer’s instructions. The protein lysates were separated on 4–12% NuPAGE Bis-Tris gels (Invitrogen) and electrotransferred to 0.2 µm PVDF (polyvinylidene difluoride) membranes (Bio-Rad). The membranes were probed with rabbit anti-Non-phospho β-catenin (1:1000, Cell signaling 4270), rabbit anti-β-catenin (1:2000, Sigma C2206), and mouse anti-GAPDH (1:800, Millipore MAB374) followed by horseradish peroxidase-conjugated anti-rabbit (Chemicon), or anti-mouse (Chemicon) antibodies. The blots were detected with ECL-Plus Western Blotting Substrate (Thermo Fisher) (See Supplementary Fig. 5). As noted in the Millipore data sheet, GAPDH resides in both the cytosol and nucleus, where GAPDH is translocated to the nucleus when cells respond to the initial stages of apoptosis or oxidative stress.

### Real-time quantitative PCR (RT-qPCR)

For each biological replicate, total RNA was extracted from HEK293T and reverse-transcribed into cDNA with oligo(dT) primers (Thermo Fisher) and Superscript III reverse transcriptase (Life Technologies). RT-qPCR was performed on a LightCycler 96 (Roche) for genes of interest with Gapdh as the housekeeping gene. Ct values for genes were averaged from three technical replicates. Amplification primers are purchased from Taqman probe: *GAPDH* (Hs02758991), *AXIN2* (Hs00610344), *CCND1* (Hs00765553), *CDKN1A* (Hs00355782), and *FGF20* (Hs00173929).

### Plasmid transfection into cell lines

HEK293T cells were seeded on 48-well collagen-coated BioCoat plates (Corning) in an antibiotic-free medium. After 12 h, HEK293T cells were transfected at ~70% confluency. For BE3 or HDR-CORRECT plasmid transfection, 750 ng of editing agent plasmid and 250 ng of sgRNA plasmids, with or without ssDNA (0, 0.7, 1.3, 2.7 µg) were transfected using 1.5 µl of Lipofectamine 2000 (Thermo Fisher) per well. For Wnt activity, unless otherwise noted, 200 ng Topflash, 20 ng Renilla, 750 ng of BE and 250 ng of sgRNA expression plasmids were transfected using 1.5 µl of Lipofectamine 2000 (Thermo Fisher) per well according to the manufacturer’s protocol. Balancing pUC19 plasmid (New England Biolabs) was transfected to make constant total DNA amount across conditions. For GUIDE-seq, 500 ng of Cas9, 250 ng of sgRNA encoding plasmids, and 100 pmol dsODN were transfected into NIH/3T3 cells using LONZA 4D-Nucleofector with the EN-158 program according to the manufacturer’s protocols.

### Protein transfection into cell lines

HEK293T cells were seeded on 48-well collagen-coated BioCoat plates (Corning) in 250 µl of an antibiotic-free medium. After 12 h, HEK293T cells were transfected at ~70% confluency. Base editor protein was incubated with 1.1 times molar excess of the necessary sgRNA at room temperature for 5 min. In parallel, Cas9 protein was incubated with 1.1 times molar excess of sgRNA and different specified molar of ssDNA at room temperature. We observed that higher sgRNA concentration with constant BE3 concentration did not increase base editing efficiency by HTS in vitro. The complex was then incubated with 1.5 µl Lipofectamine 2000 (Thermo Fisher) and transfected according to the manufacturer’s protocol for plasmid delivery. BE3 and Cas9 protein were added to a final concentration of 200 nM (based on a total well volume of 275 µl).

### High-throughput sequencing

Genomic DNA was isolated using the Agencourt DNAdvance Genomic DNA Isolation Kit (Beckman Coulter) according to the manufacturer’s instructions. First DNA amplification was performed by quantitative PCR with Phusion U Hot Start and SYBR Gold Nucleic Acid Stain (Thermo Fisher) to the top of the linear range. PCR products were purified using RapidTips (Diffinity Genomics). The second PCR was performed to attach Indexing Adapters (Illumina). The products were gel-purified and quantified using the KAPA Library Quantification Kit-Illumina (KAPA Biosystems). Samples were sequenced using a single-end read from 200–250 bases (depending on the amplicon size) on the MiSeq (Illumina) according to the manufacturer’s instructions.

### Intracochlear delivery of ribonucleoprotein

Intracochlear delivery was performed in post-natal day one (P1) CD1 mice or floxP-tdTomato mice as described previously^61^. Briefly, mice were anaesthetized by lowering the body temperature before the surgical procedure. A postauricular incision was made near the right ear, and the bulla was lifted to expose the cochlea. BE3 protein (57.7 μM stock concentration) was pre-complexed with the sgRNA or fluorescein–sgRNA (100 μM stock concentration) in a 1:1.1 molar ratio and then mixed with Lipofectamine 2000 (Thermo Fisher) in a 1:1 volumetric ratio. For delivery of (−30) GFP-Cre in floxP-tdTomato mice, 3 µL of 45 μM protein was mixed with 3 µL of Lipofectamine 2000. The resulting solution (1.0 µL) was injected with a glass pipette (end diameter, 5 μm) attached to a nanolitre micropump (WPI, UMP3 + Micro4 + NanoFil) at the rate of 150–200 nL min^−1^ through the cochlear capsule into scala media at the cochlear basal turn. Controls included the contralateral side of the uninjected cochlea, pups that received BE3 protein pre-mixed with an unrelated sgRNA, or pups that received sgRNA-only. After injection, the incision was closed and the mice were brought onto a heating pad to recover.

### EdU incorporation for cell proliferation

Pups received 10 µl of 5-ethynyl-2-deoxyuridine (EdU, 10 mg ml^−1^) by subcutaneous injection once daily for 5 days. EdU incorporation was detected with Click-iT EdU Imaging Kit (Invitrogen) according to the manufacturer’s instructions.

### Immunohistochemistry

The temporal bones of P7 pups with BE3 and EdU delivery were fixed with 4% paraformaldehyde at 4 °C overnight. Cochlear tissues were either dissected into apical, mid, and basal turn for whole-mounts or embedded in optimal cutting temperature medium (OCT) for frozen serial sections with cryostat (LEICA CM3050). After EdU detection, tissues were incubated with rabbit anti-MYO7a (1:500, Proteus Biomedical 25-6790) and goat anti-Sox2 (1:100, Santa Cruz Biotechnology sc-17320) antibodies followed with secondary antibodies conjugated with Alexa Fluor 488, and 568 (Invitrogen A11055, A10042). Images were taken with a confocal microscopy (Leica TCS SP8) and processed with Image J (NIH).

### Tissue dissection for HTS

Three to five days after the BE3: sgRNA delivery, cochlea tissues were collected by microdissection for high-throughput sequencing. Tissues were dissected into the organ of Corti, stria vascularis and modiolus. Each tissue was further dissected into between five and ten separate pieces, and DNA extraction was performed separately for each sample, followed by high-throughput sequencing as described above. The data presented in Fig. 5 shows sequencing data resulting from the extraction of one microdissected sample from each cochlear region.

### Data analysis

Sequencing reads were demultiplexed using MiSeq Reporter (Illumina). Indel frequencies were assessed using a previously described MATLAB script^3^, which counts indels of ≥1 base occurring in a 30-base window around the BE3 nicking site. Indels were defined as detectable if there was a significant difference (Student’s two-tailed *t*-test, *p* < 0.05) between indel formation in the treated sample and untreated control. Base editing frequencies were further assessed using a previously described MATLAB script^3^. In brief, reads which did not contain insertions or deletions were aligned to an appropriate reference sequence via the Smith-Waterman algorithm. Individual bases with an Illumina quality score less than or equal to 30 were converted to the placeholder nucleotide (N). This quality threshold results in nucleotide frequencies with an expected theoretical error rate of 1 in 1000. This ensures that reads containing both base edits and indels are not counted as successful base-edits, and only analyzes the non-indel containing population of reads. To calculate the number of edited reads as a percentage of the total number of successfully generated sequencing reads, the percentage of non-indel containing edited reads as measured from the alignment algorithm were multiplied by (1- fraction of reads containing an indel).

### Data availability

High-throughput sequencing data that support the findings of this study have been deposited in the NCBI Sequence Read Archive database under Accession number SRP136325. All other data are available upon reasonable request.

## Electronic supplementary material

Supplementary Information
