# Supplementary material for: In vivo base editing of post-mitotic sensory cells
Source: Nat Commun. 2018 Jun 5;9:2184. doi: 10.1038/s41467-018-04580-3 (PMC5988727; doi:10.1038/s41467-018-04580-3)
Supplement: Supplementary file 1 — Supplementary Information [file 41467_2018_4580_MOESM1_ESM.pdf]

SUPPLEMENTARY INFORMATION

In Vivo Base Editing of Post-Mitotic Sensory Cells

Wei-Hsi Yeh, Hao Chiang, Holly A. Rees, Albert S. B. Edge, and David R. Liu

Supplementary Figure 1. C-to-T conversion efficiency at β-catenin codon 33 and corresponding Wnt activity.

Supplementary Table 1. The β-catenin S33-targeting sgRNA did not result in detectable off-target modification in murine cells.

Supplementary Figure 2. Untreated wild-type mice do not exhibit cellular reprogramming, supporting cell proliferation, or hair cell proliferation in the organ of Corti.

Supplementary Figure 3. Densitometry analysis of β-catenin western blots and RT-qPCR of Wnt-responsive genes.

Supplementary Figure 4. Localization of lipid-mediated protein delivery via intracochlear injection into the mouse inner ear.

Supplementary Figure 5. Uncropped scans of the western blots in Fig. 2b.

Supplementary Note 1. Sequences of proteins used in this study.

Supplementary Note 2. Sequences of oligonucleotides used in this study.

Supplementary Table 2. *P*-values of different treatment conditions in this study.

Supplementary References

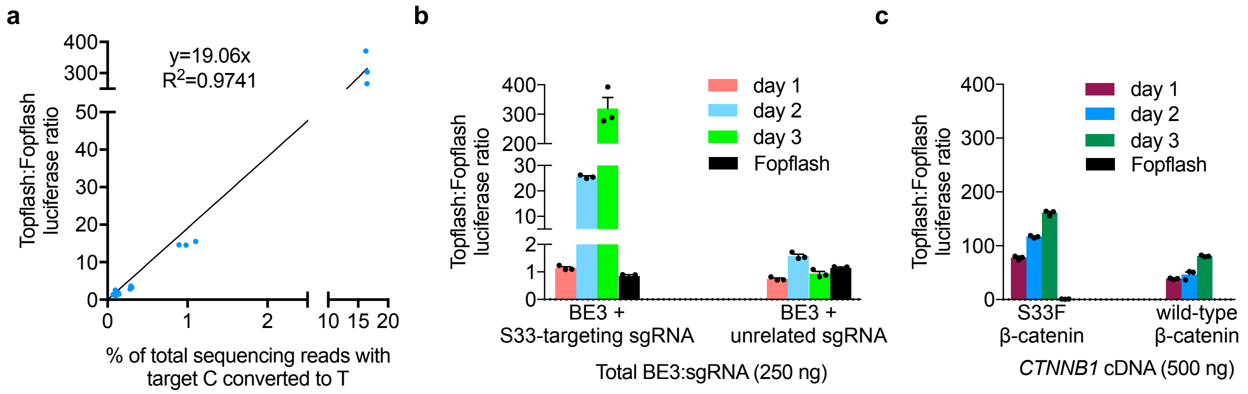

Supplementary Figure 1. C-to-T conversion efficiency at β-catenin codon 33 and corresponding Wnt activity. (**a**) The correlation between Wnt activity (Fig. 2c) and C-to-T conversion efficiency (Fig. 2d) in HEK293T cells. Linear regression analysis revealed a strong correlation with $R^2 = 0.97$. (**b, c**) Time course experiment measuring luciferase 1, 2, or 3 days after transfection with Topflash or Fopflash (a mutated negative control form of Topflash), Renilla reporter vectors, and either (b) BE3:sgRNA or (c) cDNA plasmid overexpressing F33 β-catenin or S33 β-catenin. Wnt signaling activation of TCF/LEF-mediated transcription was measured by relative luciferase activity (Topflash:Fopflash ratio), which corrects for transfection efficiencies. Values and error bars reflect mean ± S.E.M. of three technical replicates performed on three different wells.

	5’-Sequence-3’	Mismatches (MMs)	UCSC gene	Location	Indels	GUIDE-seq
On-Target	TTGGATTCTGGAATCCATTCTGG	0MMs	*Ctnnb1*	chr9:120950619	22.59%	1108
MIT-1	TTTGATTCTGGAATCCATGCAAG	2MMs		chr1:138909020	0.04%	0
MIT-2	TTGAAATCTGGAATCCATTCCAG	2MMs		chr6:53612810	0.02%	0
MIT-3	TTGGATCCTGGAATCCATGCTGA	2MMs		chr4:148110266	0.02%	0
MIT-4	TTGGTTTCTGGAATCCACTCAGG	2MMs		chrX:66244521	0.03%	0
MIT-5	GAGGATTCTGAAATCCATTCTGA	3MMs		chr10:58945312	0.02%	0
CFD-1	TGGGATATTAGAATCCATTCTGG	4MMs		chr5:33711103	0.01%	0
CFD-2	GTGGGTTCTGGGATCCATTCTGG	3MMs		chr8:41900478	0.03%	0
CFD-3	TGGGGTTTTAGAATCCATTCTGG	4MMs		chr9:42831557	0.04%	0
CFD-4	TGGGATTTGGGAATCAATTCAGG	4MMs		chr11:80023506	0.03%	0
CFD-5	ATGGATTCTGGAGACCATTCTGG	3MMs		chr7:114461205	0.02%	0

Supplementary Table 1. The β-catenin S33-targeting sgRNA did not result in detectable off-target modification in murine cells. No off-target modification was observed in NIH-3T3 cells by GUIDE-seq[1] following nucleofection of 1000 ng Cas9 plasmid and 300 ng S33-targeting sgRNA plasmid into murine NIH/3T3 cells using a LONZA 4D-Nucleofector. Potential off-target sites were also identified by computational prediction using CRISPOR[2]. Indels were assayed by HTS at the *Ctnnb1* on-target site and at each predicted off-target site. Mismatches are shown in red and PAMs are in blue. No significant off-target modification was observed either using GUIDE-seq or by high-throughput sequencing of potential off-target sites identified through two different computational prediction methods.

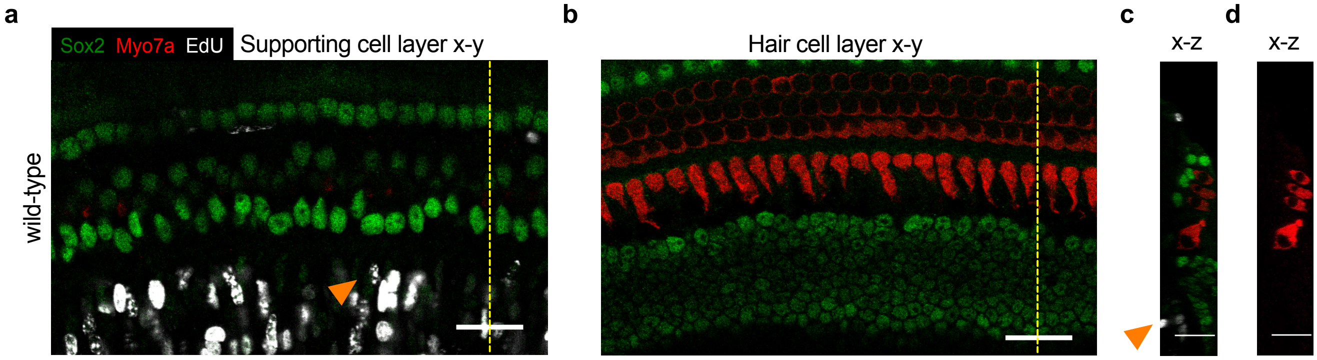

Supplementary Figure 2. Untreated wild-type mice do not exhibit cellular reprogramming, supporting cell proliferation, or hair cell proliferation in the organ of Corti. Postnatal day 2 (P2) mice received 5-ethynyl-2-deoxyuridine (EdU), which is incorporated into replicating cells, by subcutaneous injection once daily for 5 days. At postnatal day 7 (one day after the fifth EdU injection), cochlea were dissected and organ or Corti were visualized by chemical staining and immnunofluorescence. In the cochlea, Myo7a (red) is expressed in hair cells and Sox2 (green) is expressed in supporting cells. EdU (white) was used to visualize newly divided cells. (**a, b**) No EdU incorporation was observed among Sox2-positive cells or among Myo7a-positive cells, suggesting no newly divided supporting cells or hair cells. EdU-positive cells were observed among other cell types (*e.g.*, tympanic border cells, indicated by orange arrowheads in **a** and **c**). (**c, d**) An x–z plane image of (a) at the dotted yellow line, with (c) either all three channels (EdU, Myo7a, and Sox2) visualized, or (d) only Myo7a visualized. Scale bar (white) = 25 µm.

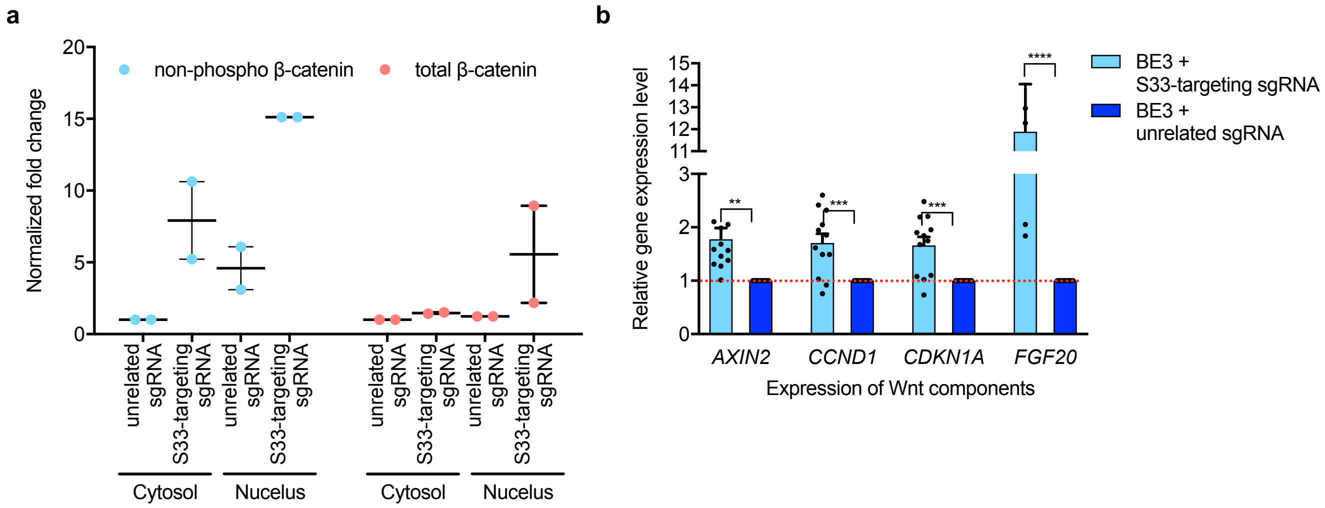

Supplementary Figure 3. Densitometry analysis of β-catenin western blots and RT-qPCR of Wnt-responsive genes. (**a**) HEK293T cells were treated with BE3 and S33-targeting sgRNA (BE3) or with unrelated sgRNA (control). Proteins extracted from each sample were separated into cytosolic and nuclear fractions. Expression levels were normalized to *GAPDH* (see Methods) and are expressed as fold change relative to control samples, which were treated with lipid only. Values and error bars reflect mean and range of two biological replicates performed on separate days. (**b**) RT-qPCR of Wnt target genes analyzed 3 days after transfection with BE3 + S33-targeting sgRNA in HEK293T cells. Fold changes were normalized to cells treated with BE3 + unrelated sgRNA. Values and error bars reflect mean ± S.E.M. of 12 samples per bar treated in three different days. ** $p \leq 0.01$, *** $p \leq 0.001$, and **** $p \leq 0.0001$ (Student's two tailed t-test).

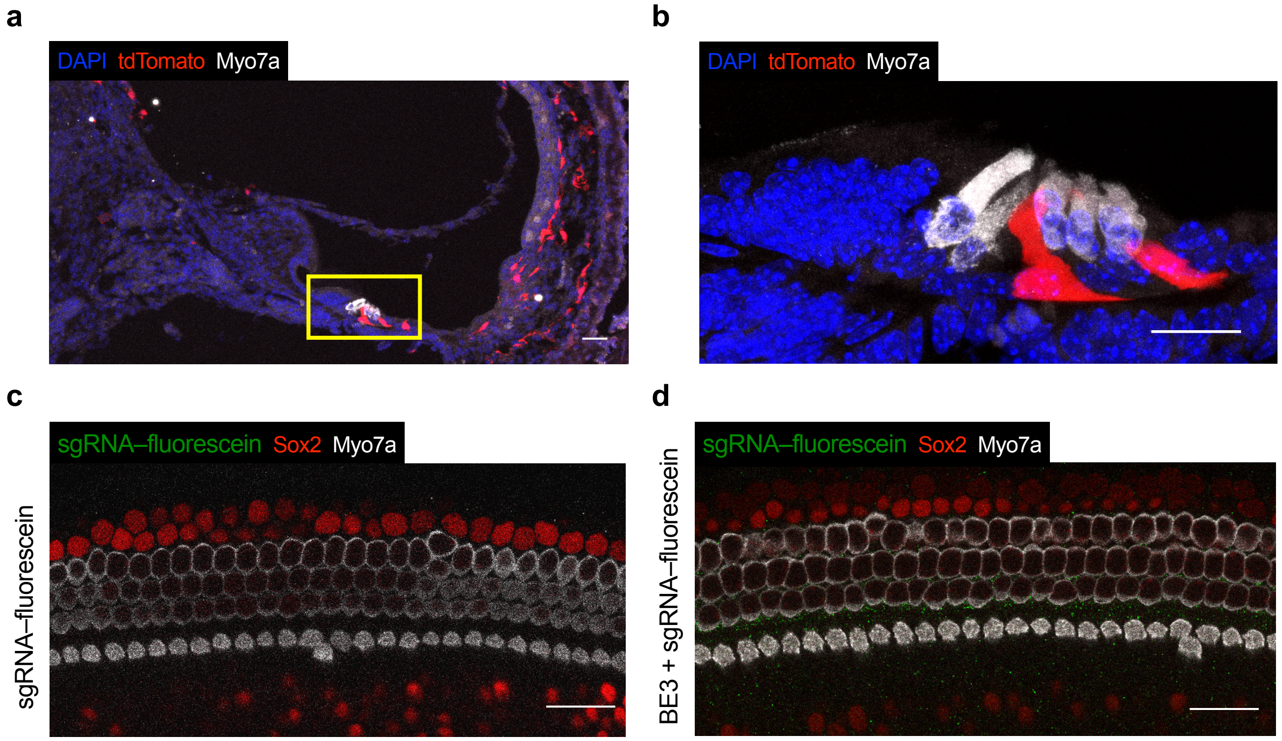

Supplementary Figure 4. Localization of lipid-mediated protein delivery via intracochlear injection into the mouse inner ear. (**a, b**) The cochlea of three postnatal day 1 (P1) mice were injected with cationic lipid (Lipofectamine 2000) complexed with (–30)GFP–Cre5,6. One day after injection, temporal bones containing the cochlea were dissected, fixed, cryoprotected with 30% sucrose, and sectioned.The organ of Corti was visualized by staining and fluorescence microscopy. A sectional view (See Fig. 4a) of one representative cochlea is shown, with Myo7a (white) denoting hair cells, DAPI (blue) denoting nuclei, and tdTomato (red) denoting Cre-mediated recombination. (a) The tdTomato signal was observed in the stria vascularis and the organ of Corti (yellow rectangle, magnified in (b)). (b) In the organ of Corti, the tdTomato signal is located in the supporting-cell region beneath the hair cells (white), demonstrating that Cre delivery is localized to the site of injection and enables targeting of supporting cells. Scale bars (white) = 20 µm. (**c, d**) The cochleas of two wild-type P1 mice were injected with cationic lipid (Lipofectamine 2000) complexed with BE3 + fluorescein-labeled S33-targeting sgRNA. Two mice injected with lipid complexed with fluorescein-labeled S33-targeting sgRNA alone. Six hours after injection, the cochleas were dissected and the organ of Corti was visualized by staining and fluorescence microscopy. A top-down view of hair cell and supporting cell layer is shown. Myo7a (white) is expressed in hair cells, Sox2 (red) is expressed in supporting cells, and sgRNA–fluorescein is shown in green. (c) No fluorescein signal near Sox2-positive cells or Myo7a-positive cells was observed in cochlea treated with sgRNA–fluorescein alone, suggesting sgRNA alone is prone to degrade *in vivo*. (d) In contrast, fluorescein signal was observed in BE3 + sgRNA–fluorescein treated cochlea in regions of Sox2-positive cells and Myo7a-positive cells, suggesting localization of injected lipid:BE3:sgRNA complexes to these regions. Scale bars (white) = 25 µm.

total β-catenin

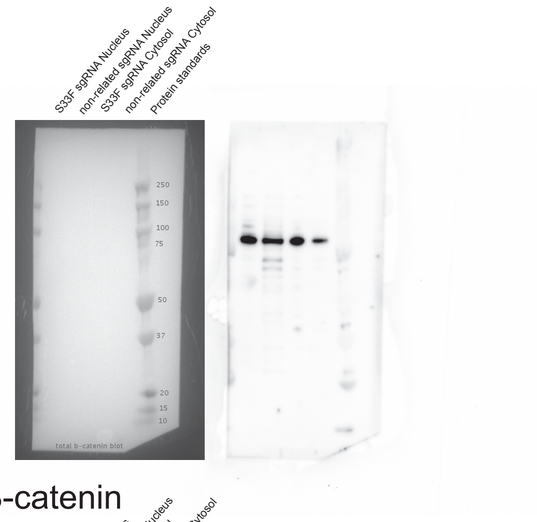

non-phospho β-catenin

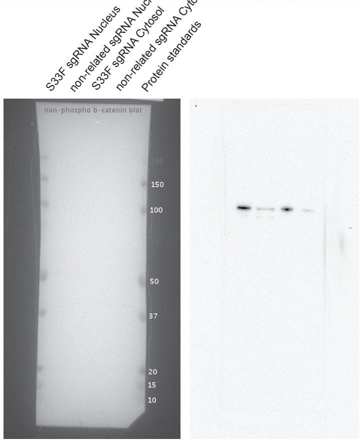

GAPDH

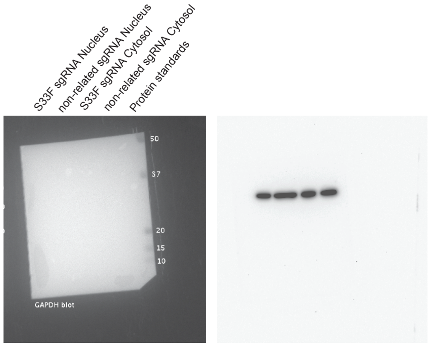

Supplementary Figure 5. Uncropped scans of the western blots in Fig. 2b. Bright field images (left) and images after exposure to ECL-Plus substrate (right) are shown after blotting with antibodies against total β-catenin (top), non-phospho β-catenin (middle), or GAPDH (bottom).

Supplementary Note 1. Sequences of proteins used in this study.
BE3 protein sequence[3]

MGSSHHHHHHSSETGPVAVDPTLRRRIEPHEFEVFFDPRELRKETCLLYEINWGGRHSIWRHTSQNTNKHVEV
NFIEKFTTERYFCPNTRCSITWFLSWSPCGECSRAITEFLSRYPHVTLFIYIARLYHHADPRNRQGLRDLISSGVTI
QIMTEQESGYCWRNFVNYSPSNEAHWPRYPHLWVRLYVLELYCIILGLPPCLNILRRKQPQLTFFTIALQSCHYQ
RLPPHILWATGLKSGSETPGTSESATPESDKKYSIGLAIGTNSVGWAVITDEYKVPSKKFKVLGNTDRHSIKKNLI
GALLFDSGETAEATRLKRTARRRYTRRKNRICYLQEIFSNEMAKVDDSFFHRLEESFLVEEDKKHERHPIFGNIV
DEVAYHEKYPTIYHLRKKLVDSTDKADLRLIYLALAHMIKFRGHFLIEGDLNPDNSDVDKLFIQLVQTYNQLFEENP
INASGVDAKAILSARLSKSRRLENLIAQLPGEKKNGLFGNLIALSLGLTPNFKSNFDLAEDAKLQLSKDTYDDDLD
NLLAQIGDQYADLFLAAKNLSDAILLSDILRVNTEITKAPLSASMIKRYDEHHQDLTLLKALVRQQLPEKYKEIFFDQ
SKNGYAGYIDGGASQEEFYKFIKPILEKMDGTEELLVKLNREDLLRKQRTFDNGSIPHQIHLGELHAILRRQEDFY
PFLKDNREKIEKILTFRIPYYVGPLARGNSRFAWMTRKSEETITPWNFEEVVDKGASAQSFIERMTNFDKNLPNE
KVLPKHSLLYEYFTVYNELTKVKYVTEGMRKPAFLSGEQKKAIVDLLFKTNRKVTVKQLKEDYFKKIECFDSVEIS
GVEDRFNASLGTYHDLLKIIKDKDFLDNEENEDILEDIVLTLTLFEDREMIEERLKTYAHLFDDKVMKQLKRRRYTG
WGRLSRKLINGIRDKQSGKTILDFLKSDGFANRNFMQLIHDDSLTFKEDIQKAQVSGQGDSLHEHIANLAGSPAIK
KGILQTVKVVDELVKVMGRHKPENIVIEMARENQTTQKGQKNSRERMKRIEEGIKELGSQILKEHPVENTQLQNE
KLYLYYLQNGRDMYVDQELDINRLSDYDVDHIVPQSFLKDDSIDNKVLTRSDKNRGKSDNVPSEEVVKKMKNYW
RQLLNAKLITQRKFDNLTKAERGGLSELDKAGFIKRQLVETRQITKHVAQILDSRMNTKYDENDKLIREVKVITLKS
KLVSDFRKDFQFYKVREINNYHHAHDAYLNAVVGTALIKKYPKLESEFVYGDYKVYDVRKMIAKSEQEIGKATAK
YFFYSNIMNFFKTEITLANGEIRKRPLIETNGETGEIVWDKGRDFATVRKVLSMPQVNIVKKTEVQTGGFSKESILP
KRNSDKLIARKKDWDPKKYGGFDSPTVAYSVLVVAKVEKGKSKKLKSVKELLGITIMERSSFEKNPIDFLEAKGY
KEVKKDLIIKLPKYSLFELENGRKRMLASAGELQKGNELALPSKYVNFLYLASHYEKLKGSPEDNEQKQLFVEQH
KHYLDEIIEQISEFSKRVILADANLDKVLSAYNKHRDKPIREQAENIIHLFTLTNLGAPAAFKYFDTTIDRKRYTSTKE
VLDATLIHQSITGLYETRIDLSQLGGDSGGSTNLSDIIEKETGKQLVIQESILMLPEEVEEVIGNKPESDILVHTAYD
ESTDENVMLLTSDAPEYKPWALVIQDSNGENKIKMLSGGSPKKKRKV

Supplementary Note 2. Sequences of oligonucleotides used in this study.

Primers used to amplify off-target genomic DNA for HTS in mouse cells

fwd_Ctnnb1_off_target_MIT_1	ACACTCTTTCCCTACACGACGCTCTTCCGATCTNNNNTCTTGTTCATGCTGTCGCTG
rev_Ctnnb1_off_target_MIT_1	TGGAGTTCAGACGTGTGCTCTTCCGATCTTTGGGTCATATGCGGGTATG
fwd_Ctnnb1_off_target_MIT_2	ACACTCTTTCCCTACACGACGCTCTTCCGATCTNNNNTGTTCTGAGAACACACCCTG
rev_Ctnnb1_off_target_MIT_2	TGGAGTTCAGACGTGTGCTCTTCCGATCTTTAATGTCAGCCAGAGCCTC
fwd_Ctnnb1_off_target_MIT_3	ACACTCTTTCCCTACACGACGCTCTTCCGATCTNNNNTGTGTCTGCAGAAGTCAAGG
rev_Ctnnb1_off_target_MIT_3	TGGAGTTCAGACGTGTGCTCTTCCGATCTCTACAGGACTCTTCAGAGGA
fwd_Ctnnb1_off_target_MIT_4	ACACTCTTTCCCTACACGACGCTCTTCCGATCTNNNNCAGAGGTGTCAAATTCCTCC
rev_Ctnnb1_off_target_MIT_4	TGGAGTTCAGACGTGTGCTCTTCCGATCTTCAGTGATTGAGTCTTGCCC
fwd_Ctnnb1_off_target_MIT_5	ACACTCTTTCCCTACACGACGCTCTTCCGATCTNNNNGTCCTCAGTTCCACAGATTC
rev_Ctnnb1_off_target_MIT_5	TGGAGTTCAGACGTGTGCTCTTCCGATCTTCTGTTCATGGCCAGCTTGA
fwd_Ctnnb1_off_target_CFD_1	ACACTCTTTCCCTACACGACGCTCTTCCGATCTNNNNAAACCATAGGAGGAGAGTGC
rev_Ctnnb1_off_target_CFD_1	TGGAGTTCAGACGTGTGCTCTTCCGATCTGGTGCAGAAGTATAGGGAAG
fwd_Ctnnb1_off_target_CFD_2	ACACTCTTTCCCTACACGACGCTCTTCCGATCTNNNNCAACTGATAAGACTTGCCTTGC
rev_Ctnnb1_off_target_CFD_2	TGGAGTTCAGACGTGTGCTCTTCCGATCTCAAGAAAAAGTGTTTTGAGGGC
fwd_Ctnnb1_off_target_CFD_3	ACACTCTTTCCCTACACGACGCTCTTCCGATCTNNNNTAGTTCAAGCTGGTCTGTGG
rev_Ctnnb1_off_target_CFD_3	TGGAGTTCAGACGTGTGCTCTTCCGATCTATCTGTGCTTCAGAGCTACC
fwd_Ctnnb1_off_target_CFD_4	ACACTCTTTCCCTACACGACGCTCTTCCGATCTNNNNATCCAAGATCCCTAGGAACC
rev_Ctnnb1_off_target_CFD_4	TGGAGTTCAGACGTGTGCTCTTCCGATCTAAAGATGAGGTGTGTGTGGC
fwd_Ctnnb1_off_target_CFD_5	ACACTCTTTCCCTACACGACGCTCTTCCGATCTNNNNTCTTCCCATCTCCTTAGCTCTG
rev_Ctnnb1_off_target_CFD_5	TGGAGTTCAGACGTGTGCTCTTCCGATCTTGTAAGAATATTAGGGGCAGGG

Primers used to amplify on-target genomic DNA for HTS

fwd_CTNNB1_on_target	ACACTCTTTCCCTACACGACGCTCTTCCGATCTNNNNATTTGATGGAGTTGGACATGGCC
rev_CTNNB1_on_target	TGGAGTTCAGACGTGTGCTCTTCCGATCTCCAGCTACTTGTTCTTGAGTGAAGG
fwd_Ctnnb1_on_target	ACACTCTTTCCCTACACGACGCTCTTCCGATCTNNNNTGGAGCCGGACAGAAAAGC
rev_Ctnnb1_on_target	TGGAGTTCAGACGTGTGCTCTTCCGATCTCTTCTTCCTCAGGGTTGCCC

Primers used for generating PCR products prior to T7 RNAP transcription of sgRNAs

rev_sgRNA_T7: used in all cases	AAAAAAAGCACCGACTCGGTGCCAC

fwd_sgRNA_T7_Ctnnb1	TAATACGACTCACTATAGGTTGGATTCTGGAATCCATTTGTTTTAGAGCTAGAAATAGCA
fwd_sgRNA_T7_CTNNB1	TAATACGACTCACTATAGGCTGGACTCTGGAATCCATTCGTTTTAGAGCTAGAAATAGCA

Primers used for generating sgRNA transfection plasmids

The pFYF1320 plasmid was used as template as previously described[4]. The sequence of other sgRNA plasmids was previously reported

rev_sgRNA_plasmid	GGTGTTTCGTCCTTTCCACAAG
fwd_CTNNB1	GAATGGATTCCAGAGTCCAGGTTTTAGAGCTAGAAATAGCAAGTTAAAATAAGGC
fwd_Ctnnb1	TTGGATTCTGGAATCCATTCGTTTTAGAGCTAGAAATAGCAAGTTAAAATAAGGC

Sequences of DNA strands for in vitro deaminase assays

fwd_Ctnnb1_substrate	ACGTAAACGGCCACAA TACTTGGATTCTGGAATCCATTCTGG GCATCTTCTTCAAGGACG
rev_Ctnnb1_substrate	CGTCCTTGAAGAAGATGC CCAGAATGGATTCCAGAATCCAAGTA TTGTGGCCGTTTACGT

P values (student's two-tailed t-test) for comparisons between listed treatments					
		Specificity ratio	Top/Fop activity	Base-editing	Signif.
Fig. 1d	plasmid CORRECT HDR vs BE3	<0.0001			****
Fig. 2a	plasmid wild-type CTNNB1 vs plasmid mutant CTNNB1		<0.0001		****
Fig. 2c	plasmid BE3:S33F sgRNA (62.5 ng) vs BE3:unrelated sgRNA (1000 ng)		0.1166		ns
Fig. 2c	plasmid BE3:S33F sgRNA (125 ng) vs BE3:unrelated sgRNA sgRNA (1000 ng)		0.0346		*
Fig. 2c	plasmid BE3:S33F sgRNA (250 ng) vs BE3:unrelated sgRNA sgRNA (1000 ng)		0.0006		***
Fig. 2c	plasmid BE3:S33F sgRNA (500 ng) vs BE3:unrelated sgRNA sgRNA (1000 ng)		<0.0001		****
Fig. 2c	plasmid BE3:S33F sgRNA (1000 ng) vs BE3:unrelated sgRNA (1000 ng)		0.0005		***
Fig. 2d	plasmid BE3:S33F sgRNA (62.5 ng) vs BE3:unrelated sgRNA (1000 ng)			0.0037	**
Fig. 2d	plasmid BE3:S33F sgRNA (125 ng) vs BE3:unrelated sgRNA (1000 ng)			0.0023	**
Fig. 2d	plasmid BE3:S33F sgRNA (250 ng) vs BE3:unrelated sgRNA (1000 ng)			<0.0001	****
Fig. 2d	plasmid BE3:S33F sgRNA (500 ng) vs BE3:unrelated sgRNA (1000 ng)			<0.0001	****
Fig. 2d	plasmid BE3:S33F sgRNA (1000 ng) vs BE3:unrelated sgRNA (1000 ng)			<0.0001	****
Fig. 3d	RNP CORRECT HDR vs BE3	0.0009			***

Supplementary Table 2. *P*-values of different treatment conditions in this study. *P*-values were calculated using the Student's two tailed t-test as described in the Methods section. ns = p > 0.05, * p ≤ 0.05, ** p ≤ 0.01, *** p ≤ 0.001, and **** p ≤ 0.0001.

Supplementary References

1. Tsai, S. Q. et al. GUIDE-seq enables genome-wide profiling of off-target cleavage by CRISPR-Cas nucleases. Nature biotechnology **33**, 187-197, doi:10.1038/nbt.3117 (2015).
2. Haeussler, M. et al. Evaluation of off-target and on-target scoring algorithms and integration into the guide RNA selection tool CRISPOR. *Genome Biology* **17**, 148 (2016).
3. Rees, H.A. et al. Improving the DNA specificity and applicability of base editing through protein engineering and protein delivery. *Nature Communications* **8**, 15790 (2017).
4. Komor, A.C., Kim, Y.B., Packer, M.S., Zuris, J.A. & Liu, D.R. Programmable editing of a target base in genomic DNA without double-stranded DNA cleavage. Nature **533**, 420-424 (2016).
